# Recent advances in immunohistochemical and molecular profiling for differential diagnosis between giant cell-rich lesions and tenosynovial giant cell tumors

**DOI:** 10.3389/fonc.2024.1511127

**Published:** 2025-01-09

**Authors:** Jiro Ichikawa, Tomonori Kawasaki, Satoshi Ochiai, Masanori Wako, Tetsuo Hagino, Kaoru Aoki, Kojiro Onohara

**Affiliations:** ^1^ Department of Orthopaedic Surgery, Interdisciplinary Graduate School of Medicine, University of Yamanashi, Yamanashi, Japan; ^2^ Department of Pathology, Saitama Medical University International Medical Center, Saitama, Japan; ^3^ Department of Orthopaedic Surgery, National Hospital Organization (NHO) Kofu National Hospital, Yamanashi, Japan; ^4^ Physical Therapy Division, School of Health Sciences, Shinshu University, Nagano, Japan; ^5^ Department of Radiology, Interdisciplinary Graduate School of Medicine, University of Yamanashi, Yamanashi, Japan

**Keywords:** giant cell-rich tumors, tenosynovial giant cell tumor, aneurysmal bone cyst, giant cell tumor of the bone, immunohistochemistry, molecular confirmation, differential diagnosis

## 1 Introduction

We read the paper by Wang et al. entitled, “A Case Report of Diffuse-type Tenosynovial Giant Cell Tumor as a Calcaneus Mass: A Diagnostic Challenge” with much interest (1). First of all, we would like to congratulate the authors with the publication of this challenging and interesting case. One of the interesting and challenging aspects of this case is that it was very difficult to determine whether the tumor originated from the bone or soft tissue, and the images suggested that the primary tumor origin was the bone, as described in their paper, which appears to be a diagnostic imaging limitation. If the tumor had originated from the bone, the most important differential diagnosis would have been giant cell-rich lesions, and we would like to discuss here that immunohistochemistry (IHC) and fluorescence *in-situ* hybridization (FISH) findings are particularly important in differentiating between this type of tumors and tenosynovial giant cell tumors (TSGCTs).

## 2 Discussion

### 2.1 Image assessment

First, the X-ray and computed tomography (CT) imaging findings suggested the presence of a benign bone tumor because of its soap-bubble appearance and sclerotic rim, and it was reasonable to assume that the medial bone defect was due to a pathological fracture by a bone tumor. Magnetic resonance imaging findings—contrast images were unfortunately not available—were suggestive of both cystic and solid tumors. Blooming on T2*WI is a useful finding in TSGCTs, but it is not highly sensitive (2). Therefore, we agree with aneurysmal bone cyst (ABC) as a first differential diagnosis as reported in the paper, and further, we consider the possibility of giant cell tumor of the bone (GCTB), which often causes a pathological fracture. The diagnosis of a soft tissue tumor that has invaded the bone is very challenging in any case.

### 2.2 Histopathological findings in differential diagnosis

ABC and GCTB are giant cell-rich tumors, which are often challenging to diagnose based on imaging and histopathology. Interestingly, TSGCT, although not a bone tumor, is a giant cell-rich tumor. In what follows, we focus on the molecular features of ABC and immunohistochemical features of GCTB.

#### 2.2.1 Ubiquitin-specific peptidase 6 rearrangement as a marker of ABC

Specific and useful IHC markers for the diagnosis of ABC are lacking (3). Therefore, it has often been difficult to differentiate between ABC and tumors presenting with secondary ABC and solitary bone cysts (SBCs). Chromosomal rearrangement of *USP6* has been found in ABC, and USP6 activation has a wide range of effects, including osteogenesis, osteolysis, inflammation, angiogenesis, and tumorigenesis (4). CDH11 is a fusion partner of USP6 (~30%), in addition to RUNX2, COL1A1, and 50 other fusion partners (4). Among these 50 fusion partners, 30 have been reported in ABCs (4, 5). The detection rate of USP6 rearrangement based on molecular testing is approximately 70%, but it should be noted that this may be an underestimation due to demineralization (4, 5). In a recent report, *USP6* rearrangement was found not only in ABCs but also in nodular fasciitis, myositis ossificans, and fibroma of the tendon sheath in soft tissue tumors, which are considered USP6-associated neoplasms (4). SBCs, which occasionally occur in the calcaneus, are often difficult to distinguish from ABCs, and NFATC2 fusion is often detected in SBCs, which may be helpful in the diagnosis (6). Furthermore, the fact that USP6 rearrangement is generally absent in tumors with secondary ABCs and telangiectatic osteosarcoma is very useful because the differential diagnosis of these tumors and ABC has been problematic (4, 5). There are currently no reports on whether a diagnosis based on USP6 expression according to IHC is possible (4).

#### 2.2.2 H3.3G34W as an IHC marker of GCTB

The rate of *H3F3A* (known as H3.3A) somatic mutation in GCTB is reportedly as high in 92.4% (7). Amary et al. reported that *H3F3A* mutation involving glycine 34 occurs in 96% of GCTB cases; particularly, p.Gly34Trp (G34W) mutation occurs in 97% of GCTB cases and G34L, V, and M variants in the remaining cases (8). They used an H3.3G34W antibody for IHC for the diagnosis of GCTB; the antibody had a positivity rate as high as 90.6%, confirming its usefulness for diagnosis (8). In addition, the antibody has near 100% specificity (9). However, caution is necessary for malignant bone tumors because of the possibility of positive staining in cases of malignant GCTB and osteosarcoma with osteoclast-rich component (8). Interestingly, denosumab-treated GCTB showed less H3.3G34W antibody positivity in IHC than conventional GCTB (9). However, negative staining was reported in ABC, chondroblastoma, non-ossifying fibroma, fibrous dysplasia, and TSGCT (10). Besides H3.3G34W IHC, the usefulness of p63 IHC has been reported, with positivity rates of 96.8% in GCTB, 22.2% in ABC, and 0% in TSGCT (11).

#### 2.2.3 IHC markers of TSGCT

Colony-stimulating factor 1 (CSF1) has recently received attention and been used as an IHC marker of TSGCT, with positivity rates of 79.5% in localized TSGCT and 75% in diffuse TSGCT, averaging 77% (12). In contrast, GCTB and ABC, as well as sarcomas, including undifferentiated pleomorphic sarcoma, leiomyosarcoma, and myxofibrosarcoma, stained negative in all cases. In addition, FISH confirmed the rearrangement of CSF1 in CSF-1-positive TSGCT. CSF1 in TSGCT is not only a diagnostic marker but also a therapeutic target. A recent phase 3 trial reported the clinical efficacy of the CSF1R inhibitor vimseltinib in TSGCT (13). In addition, the efficacy of clusterin IHC in diagnosing TSGCT has been reported (14). Cytoplasmic clusterin expression was detected in large mononuclear cells in both diffuse and localized TSGCTs (14).

#### 2.2.4 Other differential diagnosis

Finally, we showed the features of other giant cell rich tumors, including chondroblastoma, langerhans cell histiocytosis, non-ossifying fibroma/benign fibrous histiocytoma, xanthogranulomatous epithelial tumor and brown tumor of hyperparathyroidism, in brief. In the chondroblastoma, H3K36M showed diffuse nuclear expression in almost all cases, and DOG1 and SOX9, although not specific, may be focally positive (15). In langerhans cell histiocytosis, CD1a, CD207 (langerin), S100, CD68, and HLA-DR were positive (16). Non-ossifying fibroma/benign fibrous histiocytoma, *KRAS* and *FGFR1* mutations were confirmed (17). Xanthogranulomatous epithelial tumors in IHC showed diffuse positivity with the AE1/AE3 keratin antibodies, and more variably positivity with the OSCAR antibody, CK7, and high-molecular-weight keratins (18). In brown tumors, KRAS mutation was reported (19).

## 3 Conclusion

Tumors that occur at atypical sites or are difficult to identify are clinically very valuable and provide physicians with new insights. To enhance the value of such cases, the diagnosis must be made carefully and appropriately. Especially in the field of bone and soft-tissue tumors, an accurate diagnosis cannot be made based on imaging alone, but imaging findings must be combined with pathological findings. Guided by morphological hematoxylin and eosin-stained specimen sections, a comprehensive diagnosis must be made using IHC and molecular testing.
